# MICAgen Mice Recapitulate the Highly Restricted but Activation-Inducible Expression of the Paradigmatic Human NKG2D Ligand MICA

**DOI:** 10.3389/fimmu.2020.00960

**Published:** 2020-06-04

**Authors:** Younghoon Kim, Christina Born, Mathieu Bléry, Alexander Steinle

**Affiliations:** ^1^Institute for Molecular Medicine, Goethe-University Frankfurt am Main, Frankfurt am Main, Germany; ^2^Science & Innovation Division, Innate Pharma Research Laboratories, Innate Pharma, Marseille, France; ^3^Frankfurt Cancer Institute, Frankfurt am Main, Germany

**Keywords:** MICA, NKG2D, cancer immunobiology, soluble MICA, mouse model

## Abstract

NKG2D is a potent activating immunoreceptor expressed on nearly all cytotoxic lymphocytes promoting their cytotoxicity against self-cells expressing NKG2D ligands (NKG2DLs). NKG2DLs are MHC class I-like glycoproteins that usually are not expressed on “healthy” cells. Rather, their surface expression is induced by various forms of cellular stress, viral infection, or malignant transformation. Hence, cell surface NKG2DLs mark “dangerous” cells for elimination by cytotoxic lymphocytes and therefore can be considered as “kill-me” signals. In addition, NKG2DLs are up-regulated on activated leukocytes, which facilitates containment of immune responses. While the NKG2D receptor is conserved among mammals, NKG2DL genes have rapidly diversified during mammalian speciation, likely due to strong selective pressures exerted by species-specific pathogens. Consequently, NKG2DL genes are not conserved in man and mice, although their NKG2D-binding domains maintained structural homology. Human NKG2DLs comprise two members of the MIC (MICA/MICB) and six members of the ULBP family of glycoproteins (ULBP1–6) with MICA representing the best-studied human NKG2DLs by far. Many of these studies implicate a role of MICA in various malignant, infectious, or autoimmune diseases. However, conclusions from these studies often were limited in default of supporting *in vivo* experiments. Here, we report a MICA transgenic (MICAgen) mouse model that replicates central features of human MICA expression and function and, therefore, constitutes a novel tool to critically assess and extend conclusions from previous *in vitro* studies on MICA. Similarly to humans, MICA transcripts are broadly present in organs of MICAgen mice, while MICA glycoproteins are barely detectable. Upon activation, hematopoietic cells up-regulate and proteolytically shed surface MICA. Shed soluble MICA (sMICA) is also present in plasma of MICAgen mice but affects neither surface NKG2D expression of circulating NK cells nor their functional recognition of MICA-expressing tumor cells. Accordingly, MICAgen mice also show a delayed growth of MICA-expressing B16F10 tumors, not accompanied by an emergence of MICA-specific antibodies. Such immunotolerance for the xenoantigen MICA ideally suits MICAgen mice for anti-MICA-based immunotherapies. Altogether, MICAgen mice represent a valuable model to study regulation, function, disease relevance, and therapeutic targeting of MICA *in vivo*.

## Introduction

Expression of the activating immunoreceptor NKG2D facilitates immunorecognition and elimination of “harmful,” activated, or stressed cells by cytotoxic lymphocytes (1–3). NKG2D ligates several MHC class I-related glycoproteins, which are usually not expressed on “healthy” cells but inducibly expressed upon viral infection, malignant transformation, or cellular activation (2, 4). In humans, such inducible NKG2D ligands (NKG2DLs) include two members of the MIC (MICA and MICB) and six members of the ULBP family (ULBP1–6) of MHC class I-related glycoproteins (1, 4, 5). Although all MIC and ULBP molecules share MHC class I-like α1α2 superdomains, only MICA/B molecules contain an additional immunoglobulin-like α3 ectodomain alike MHC class I molecules (4, 6). Infection of human cells with various types of viruses, including herpesviruses and adenoviruses, strongly induces NKG2DL expression, which often is counteracted by NKG2DL-specific viral immunoevasins (7–10). Also, activation of hematopoietic cells can induce a pronounced surface expression of NKG2DLs (11–13), which has been suggested to contribute to the modulation and control of immune responses (11, 14). A functional relevance of NKG2DLs has also been implicated in many different autoimmune diseases (15). But foremost, the broad expression of MIC and ULBP molecules by tumor cell lines and human tumors in contrast to the corresponding non-malignant tissues has spawned most interest in harnessing the NKG2D/NKG2DL system for cancer immunotherapy (16–18). Specifically, MICA is expressed by many different tumors and therefore constitutes an attractive target for immunotherapy (16, 19). However, expression of NKG2DLs by established human tumors also suggests that NKG2D-mediated cancer immunosurveillance is paralyzed in such tumors. Several immune escape mechanisms sabotaging NKG2D-mediated tumor surveillance have been reported, including down-regulation of NKG2D by cytokines such as TGF-β (18, 20) or tumor-derived soluble NKG2DL (sNKG2DL) as well as of down-regulation of NKG2DL expression by proteolytic shedding, miRNA, or cytokines (20–23). Hence, following the excitement created by early tumor rejection studies in mice (24, 25), subsequent reports on various NKG2D immune escape mechanisms in human cancer dampened this enthusiasm to some extent. A major problem in the field of disease relevance of human NKG2DLs and their therapeutic targeting is that human NKG2DLs are not conserved in mice. Therefore, many hypotheses on the relevance and pathogenic role of human NKG2DLs in malignant and autoimmune diseases derived from *in vitro* studies or correlative studies cannot easily be verified or falsified in appropriate mouse models. This includes, for example, hypotheses on the functional relevance of sNKG2DL in malignant disease or on NKG2DL expression by the intestinal epithelium for gastrointestinal diseases. Here, we present a transgenic mouse model for the paradigmatic human NKG2DL MICA, which replicates central aspects of MICA expression reported for the human situation. We anticipate that this mouse model will allow insightful studies on the regulation of MICA expression and functional relevance of MICA in immune responses and disease settings *in vivo*. As a first example, we report that surface MICA expression can be induced by activation of splenocytes accompanied by shedding of soluble MICA (sMICA), which is also detectable in plasma of MICAgen mice and apparently does not systemically down-regulate NKG2D expression.

## Materials and Methods

### Mice

MICA transgenic mice (MICAgen mice) harboring a 23.4 kb genomic fragment comprising the human MICA^*^007 gene were generated as follows: one-cell embryos of (C57BL/6 × SJL) F1/J hybrid mice were microinjected with a 23.4 kb *Xba*I/*Sal*I fragment isolated from a cosmid encompassing the entire human MICA gene (position 31,393,601 to 31,417,018 on Chr6p21 according to GRCh38.p12). The cosmid MB24/1 was isolated from a human cosmid library of human genomic DNA (26) by screening with a MICA cDNA probe. Eggs were transferred into the oviducts of B6 CBA mice, and offspring was tested for the presence of the MICA transgene using genomic DNA from tail snips. Resulting MICA transgenic mice were termed MICAgen mice and have been continuously backcrossed with C57BL/6J mice (Envigo, Horst, Netherlands) for more than 20 years at our local animal facilities before the beginning of the experiments. Non-transgenic littermates (nontgLM) of MICAgen mouse breedings were used for controls. The cosmid MB24/1 had also been transfected into mouse L-tk cells with MICA-expressing L-tk transfectants being used for cloning of the corresponding MICA cDNA by reverse transcription–PCR (RT-PCR). Transgenic mice expressing this human MICA cDNA (MICA^*^00701 allele; GenBank accession number ) under control of the H2-K^b^ promoter have been termed H2-K^b^-MICA and been described elsewhere (27, 28). Litters were tested transgenes by PCR using genomic DNA or for MICA surface expression on peripheral blood lymphocytes (PBL) by flow cytometry using anti-MICA mAb AMO1-AlexaFluor647. NKG2D-deficient (Klrk1^−/−^) mice (29) were kindly provided by Dr. Bojan Polic. Animals were maintained under specific pathogen-free conditions in the animal facilities of the University Hospital Frankfurt am Main, Germany. Animal experiments were approved by the local authorities (Regierungspräsidium Darmstadt, Germany, permit nos. FU/Anz. 1035, FU/Anz. 1037, and FU/1115) and performed in full compliance with the respective national guidelines.

### Cells

B16F10-mock and B16F10-MICA^*^001 transductants were previously described (27). B16F10 transductants were cultured in Dulbecco's modified Eagle medium (DMEM) (Sigma-Aldrich, Steinheim, Germany) supplemented with 10% fetal calf serum (FCS) (Biochrom, Berlin, Germany), 2 mM l-glutamine (Sigma-Aldrich), 100 U/ml penicillin, 100 μg/ml streptomycin (Sigma-Aldrich), 1 mM sodium pyruvate (Life Technologies, Darmstadt, Germany), and non-essential amino acids (Sigma-Aldrich). C1R cells were cultured in Roswell Park Memorial Institute (RPMI) 1640 (Sigma-Aldrich) supplemented with 10% FCS, 2 mM l-glutamine, 100 U/ml penicillin, 100 μg/ml streptomycin, 1 mM sodium pyruvate, and 1.8 mg/ml G418 (Carl Roth, Karlsruhe, Germany). For isolation of blood lymphocytes, blood was collected from hearts of euthanized mice, and cells were washed with phosphate-buffered saline (PBS) and resuspended in 5 ml of BD pharm lyse buffer (BD Biosciences, Heidelberg, Germany) for 5 min at RT for lysis of erythrocytes. After washing with PBS, lysis of erythrocytes was repeated, and cells were washed and resuspended with fluorescence-activated cell sorting (FACS) buffer (PBS, 2% FCS, 2 mM EDTA, and 0.01% sodium azide). Freshly isolated spleens were minced and passed through a 40 μm cell strainer (Greiner Bio-One, Kremsmünster, Austria). Resulting single-cell suspensions were washed with PBS, centrifuged, and subjected to lysis of erythrocytes. After another passage through a 40 μm cell strainer, cells were washed again with PBS and resuspended in FACS buffer or medium. Livers, lymph nodes, and lungs of mice were minced and passed through a 40 μm cell strainer (Greiner Bio-One) to obtain a single-cell suspension. After washing with PBS, liver cells were resuspended with BD pharm lyse buffer (BD Biosciences) and incubated for 5 min at RT to eliminate erythrocytes. Subsequently, liver cells, as well as single-cell suspensions of lymph nodes and lungs, were washed with PBS and resuspended in FACS buffer for flow cytometry.

### *In vitro* Assays With Splenocytes

For *in vitro* assays, freshly isolated mouse splenocytes (see above) were resuspended at 1 × 10^6^ cells/ml in complete RPMI 1640 supplemented with 10% FCS, 2 mM l-glutamine, 1 mM sodium pyruvate, 100 U/ml penicillin, 100 μg/ml streptomycin, 50 μM β-mercaptoethanol, and non-essential amino acids. Induction of cell surface expression of MICA was assessed upon exposure to either 10 μg/ml lipopolysaccharide (LPS) or to a combination of 50 ng/ml phorbol myristate acetate (PMA) and 1 μM ionomycin (PMA/I) (all from Sigma-Aldrich). In some experiments, splenocytes were continuously exposed to either PMA/I or LPS for 8 to 72 h before analysis. In other experiments, splenocytes were short-term treated with PMA/I for either 0.5 h or 2 h. Afterwards, splenocytes were repeatedly washed with PBS and *in vitro* cultures continued in the absence of PMA/I for up to 96 h. To assess modulation NKG2D surface expression by membrane-bound MICA, fresh single-cell suspensions of spleens from nontgLM, MICAgen, and H2-K^b^-MICA mice were prepared in medium as described above. NK cells were purified from spleens of nontgLM using the mouse NK cell isolation kit II (Miltenyi Biotec, Bergisch Gladbach, Germany) according to the manufacturer's protocol and labeled with carboxyfluorescein succinimidyl ester (CFSE) by incubation for 20 min with 0.5 μM CFSE (Thermo Fisher Scientific, Waltham, MA). After washing, CFSE-labeled NK cells were co-cultured with splenocyte cultures (at ~1 × 10^6^ cells/ml) for 24 h in a 24-well plate and subsequently analyzed for their NKG2D surface expression. To assess modulation of NKG2D surface expression by shed sMICA, splenocyte cultures (1 × 10^6^ cells/ml) were seeded into wells of a 24-well plate and costar transwell permeable supports (24 well, 1 μm pore size) (Corning, Corning, NY) containing CFSE-labeled NK cells (5 × 10^6^ cells/ml) placed atop. NKG2D surface expression of CFSE-labeled NK cells was determined by flow cytometry after 24 h *in vitro* culture at 37°C.

### Flow Cytometry

Cells were harvested, washed twice with FACS buffer, and stained with 10 μg/ml biotinylated mAb BAMO1 (anti-MICA/B) or mAb AMO1 (anti-MICA), generated in our laboratory as previously described (9, 30, 31), for 20 min at 4°C. Then, cells were washed with FACS buffer and stained with 2.5 μg/ml fluorochrome-conjugated streptavidin (BioLegend, San Diego, CA) for 20 min at 4°C. After additional washing, flow cytometry analyses were performed using a FACS Canto II (BD Biosciences) and data analyzed using FlowJo (Tree Star, Ashland, OH). For cytometric analyses of mouse cells isolated from various organs, single-cell suspensions were first incubated with anti-mCD16/32 (clone 93, BioLegend) for blocking Fc receptors for 20 min at 4°C. Cells were then stained with fluorochrome-conjugated antibodies for 20 min at 4°C: anti-mCD45.2-PerCP (clone 104, BioLegend), anti-mCD3ε-PE/Cy7 (clone 145-2C11, BioLegend), anti-mCD3-PerCP (clone 145-2C11, BioLegend), anti-mCD3-AF647 (clone 145-2C11, BioLegend), anti-mCD11b-PE/Cy7 (clone M1/70, Invitrogen, Carlsbad, CA), anti-mCD8α-APC/Cy7 (clone 53-6.7, BioLegend), anti-mCD4-AF647 (clone GK1.5, BioLegend), anti-mγδTCR-PE (clone GL3, BioLegend), anti-mNK1.1-APC (clone PK136, Invitrogen), anti-mCD49b-APC (clone DX5, BioLegend), anti-mCD19-APC/Cy7 (clone 6D5, BioLegend), anti-Ly-6G/Ly-6C(Gr-1)-PE (clone RB6-8C5, BioLegend), anti-mNKp46-FITC (clone 29A1.4, BioLegend), anti-mNKG2D-BV421 (clone CX5, BD Biosciences), and Rat κ Isotype IgG1-BV421 (clone R3-34, BD Biosciences). For data analysis of mouse primary cells, all gatings contain singlet gating and viability staining with Fixable Viability Dye eFluor 506 (eBioscience, San Diego, CA). Soluble human and mouse NKG2D ectodomains were refolded from *Escherichia coli* inclusion bodies as previously described (9) and tetramerized with APC-conjugated streptavidin (SA-APC) (Jackson ImmunoResearch Laboratories, West Grove, PA) immediately before use. Endogenous anti-MICA antibodies in plasma of mice inoculated with B16F10-MICA tumors were detected by flow cytometry. Blood was collected prior to tumor inoculation (by facial vein puncture) and after euthanasia due to tumor growth (by cardiac puncture) and plasma stored at −20°C until use. For analysis, C1R-MICA^*^001 cells (and for control, C1R-mock cells) were incubated with mouse plasma (1:50 diluted in FACS buffer), and after repeated washing, binding mouse IgG antibodies were detected with APC-conjugated goat anti-mouse IgG antibodies (Jackson ImmunoResearch Laboratories). Specific fluorescence intensity (SFI) was calculated by subtracting the median fluorescence intensity (MFI) of the control staining from the MFI of the staining of interest.

### Reverse Transcription and Quantitative Real-Time PCR

RNA was isolated from mouse tissue samples or cells using peqGOLDTriFast (Peqlab, Erlangen, Germany). Conversion into cDNA was performed using M-MLV RT (H-) (Promega, Madison, WI) according to the manufacturer's protocol after treatment with DNase I (Promega) for 45 min at 37°C. With the use of the real-time PCR system StepOnePlus (Applied Biosystems, Waltham, MA) together with SYBR Green technology (Roche, Mannheim, Germany), abundance of MICA transcripts in the respective cDNA was assessed using the oligonucleotides: MICAEX3F: 5′-CCTTGGCCATGAACGTCAGG-3′, and MICAEX4R: 5′-CCTCTGAGGCCTCACTGCG-3′. Copy numbers were normalized with the ΔΔCt method using 18S rRNA (forward: 5′-CGGCTACCACATCCAAGGAA-3′, reverse: 5′-GCTGGAATTACCGCGGCT-3′) as previously described (30).

### ELISA

Concentrations of sMICA in cell culture supernatants or plasma were determined by modifications of the previously reported MICA-specific sandwich ELISA (30, 31). In brief, the MICA-specific mAb AMO1 (5 μg/ml) was immobilized to plates, respectively, and incubated overnight at 4°C. After blocking [7.5% bovine serum albumin (BSA) in PBS], plates were washed with 0.05% TWEEN 20. Culture supernatants and plasma were mostly diluted 1:10 (in 7.5% BSA in PBS), respectively, prior adding to the plate. After incubation and washing, bound sMICA was detected by biotinylated anti-MICA/B mAb BAMO1 (1 μg/ml) followed by horseradish peroxidase (HRP)-conjugated streptavidin (SA-HRP) (1:4,000 diluted with 3.75% BSA in PBS) (Jackson ImmunoResearch Laboratories). Plates were washed extensively before adding TMB peroxidase substrate (KPL, Gaithersburg, MD). HRP activity was stopped by adding 1 M phosphoric acid, and the absorbance was measured at 450 nm. Concentrations of anti-MICA antibodies in mouse plasma were determined by ELISA, as follows: recombinant sMICA^*^007 (5 μg/ml), purified from supernatants of sMICA^*^007-transfected 293F cells, was immobilized to plates. After blocking (7.5% BSA in PBS), plates were washed with 0.05% TWEEN 20. Plasma diluted in 7.5% BSA (in PBS) (dilution range 1:17 to 1:450) was added. MICA-specific mouse mAb AMO1 serially diluted in 7.5% BSA (in PBS) was used as a standard (range: 100 to 0.05 ng/ml). After incubation and washing, bound anti-MICA antibodies were detected by HRP-conjugated goat anti-mouse IgG (H+L) antibodies (1:10,000 diluted) (Jackson ImmunoResearch Laboratories). ELISA was completed as described above, and absorbance was measured at 450 nm.

### Immunoblotting

For immunoblot analysis, *ex vivo* stimulated splenocytes were lysed using NP-40 lysis buffer (50 mM Tris–HCl pH 8.0, 150 mM NaCl, and 1% NP-40) containing the Complete Protease Inhibitor Cocktail (Roche). For deglycosylation of proteins, lysates, or culture supernatants were treated with PNGaseF (New England BioLabs, Frankfurt, Germany) according to the manufacturer's instructions for 2 h at 37°C. Cellular lysates were fractionated by sodium dodecyl sulfate-polyacrylamide gel electrophoresis (SDS-PAGE) in a Mini-PROTEAN Tetra Cell (Bio-Rad, Munich, Germany) under reducing conditions transferred to polyvinylidene difluoride (PVDF) membranes (GE Healthcare, Munich, Germany) by semi-dry blotting. Membranes were blocked by incubating with 5% milk powder dissolved in 0.1% TBST for 2 h at RT and MICA detected with either anti-MICA/B mAb BAMO1 followed by HRP-conjugated goat anti-mouse IgG antibodies or biotinylated BAMO1 followed by SA-HRP. BAMO1 was used at 10 μg/ml. Secondary reagents were from Jackson ImmunoResearch Laboratories and used at 1:10,000 dilution. Membranes were developed by HRP-juice PLUS (PJK, Kleinblittersdorf, Germany). Membranes were stripped with ReBlot Plus Mild Antibody Stripping Solution (Merck Millipore, Billerica, MA) and stained with HRP-conjugated anti-β-actin (1:10,000 diluted, clone AC-15, Sigma-Aldrich) as loading control.

### Immunofluorescence

Tissues of mice were frozen in Tissue-Tek (Sakura, Tokyo, Japan). Cryosections (thickness: 8-10 μm) prepared with a cryomicrotome (Leica Biosystems, Nussloch, Germany) were fixed in 100% cold acetone (4°C) for 10 min. Subsequently, 100% cold acetone was added to dried sections and, after 10-min incubation, progressively replaced by 75% cold acetone and 50% acetone, in steps of 30 s. After rehydration in TBS and blocking with Biotin Blocking System (Dako, Glostrup, Denmark) according to the manufacturer's protocol, 30 μg/ml mouse IgG (Thermo Fisher Scientific) and 10 μg/ml anti-CD16/32 antibody (clone 93, BioLegend) (in 3% BSA/TBS) were added for 20 min, and finally cryosections were stained for 1 h in 3% BSA/TBS containing 5 μg/ml biotinylated BAMO1. BV421-conjugated streptavidin (SA-BV421) (1:200, BioLegend) was used for secondary staining. All sections were counterstained with SYTOX Green and, after washing, covered with ProLong Gold (both from Thermo Fisher Scientific) and a cover slip. Tissue sections were visualized using a DMI6000B microscope connected to a DFC3000G camera (Leica Microsystems, Wetzlar, Germany).

### Cytotoxicity Assay

To activate NK cells, nontgLM, MICAgen, and Klrk1^−/−^ mice were injected with 15 μg poly(I:C)/g body weight [poly(I:C); InvivoGen, San Diego, CA]. Sixteen hours later, spleens were harvested and minced and passed through a 40 μm nylon mesh, and splenocytes were washed with ice-cold PBS. Erythrocytes were removed by gradient centrifugation with Ficoll-Paque Plus (GE Healthcare), and NK cells were isolated from splenocytes using the mouse NK cell isolation kit II (Miltenyi Biotec) according to the manufacturer's protocol. B16F10 transductants were labeled with 50 μCi of ^51^Cr (PerkinElmer, Waltham, MA) for 2 h at 37°C. After washing, ^51^Cr-labeled cells were incubated for 4 h at 37°C with purified NK cells at different effector (E) to target (T) ratios. Subsequently, supernatants were mixed with OptiPhase SuperMix scintillation mixture in an IsoPlate-96 and measured with a MicroBeta2 plate counter (all from PerkinElmer). Spontaneous chromium release of target cells was always less than 17% of the maximum release of target cells lysed in 1% Triton X-100. Percentage of lysis was calculated as follows: 100 × (experimental release – spontaneous release)/(maximum release – spontaneous release). Experiments were performed in triplicates.

### Tumor Experiments

B16F10-MICA^*^001 and B16F10-mock cells were harvested using a PBS-based enzyme-free cell dissociation buffer (Life Technologies) and washed twice with PBS; 1 × 10^4^ cells (in 100 μl) were mixed with 100 μl Matrigel (Corning) and injected subcutaneously into the flank of mice using a 26-gauge needle. Tumor growth was monitored daily, and tumor size measured with a metric caliper every other day. Tumor volume (mm^3^) was calculated according to the following formula: [width of tumor (mm) × width of tumor (mm) × length of tumor (mm)]/2 = tumor volume (mm^3^). According to the regulations of the authorities, mice were euthanized when tumors exceeded ~1,300 mm^3^.

### Statistical Analysis

Statistical analyses as detailed in the figure legends were performed using Prism 7 GraphPad (GraphPad Software, San Diego, CA).

## Results

### MICA Gene Expression by MICAgen Mice

**Figure 1:**
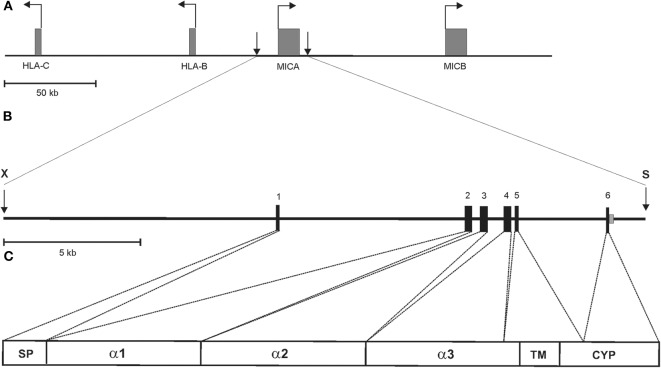
Schematic representation of the 23.4 kb MICA transgene of MICAgen mice. **(A)** Partial genomic map of the human MHC class I region on the short arm of chromosome 6 with the functional MIC genes, MICA and MICB, indicated. Horizontal arrows represent transcriptional direction of boxed genes. Vertical arrows delimit the MICA transgene. **(B)** Genomic map and location of the 23.4 kb *Xba*I (X)–*Sal*I (S) fragment from a cosmid containing the entire human MICA gene (allele MICA*00701), which was introduced into the germline of (C57BL/6 × SJL) F1/J hybrid mice. The MICA gene spans ~11.7 kb comprising six coding exons and a long first intron. Exons are boxed and protein-coding domains indicated in **(C)** with SP, signal peptide; TM, transmembrane domain; CYP, cytoplasmic domain. **(A–C)** Drawn to scale.

**Figure 2:**
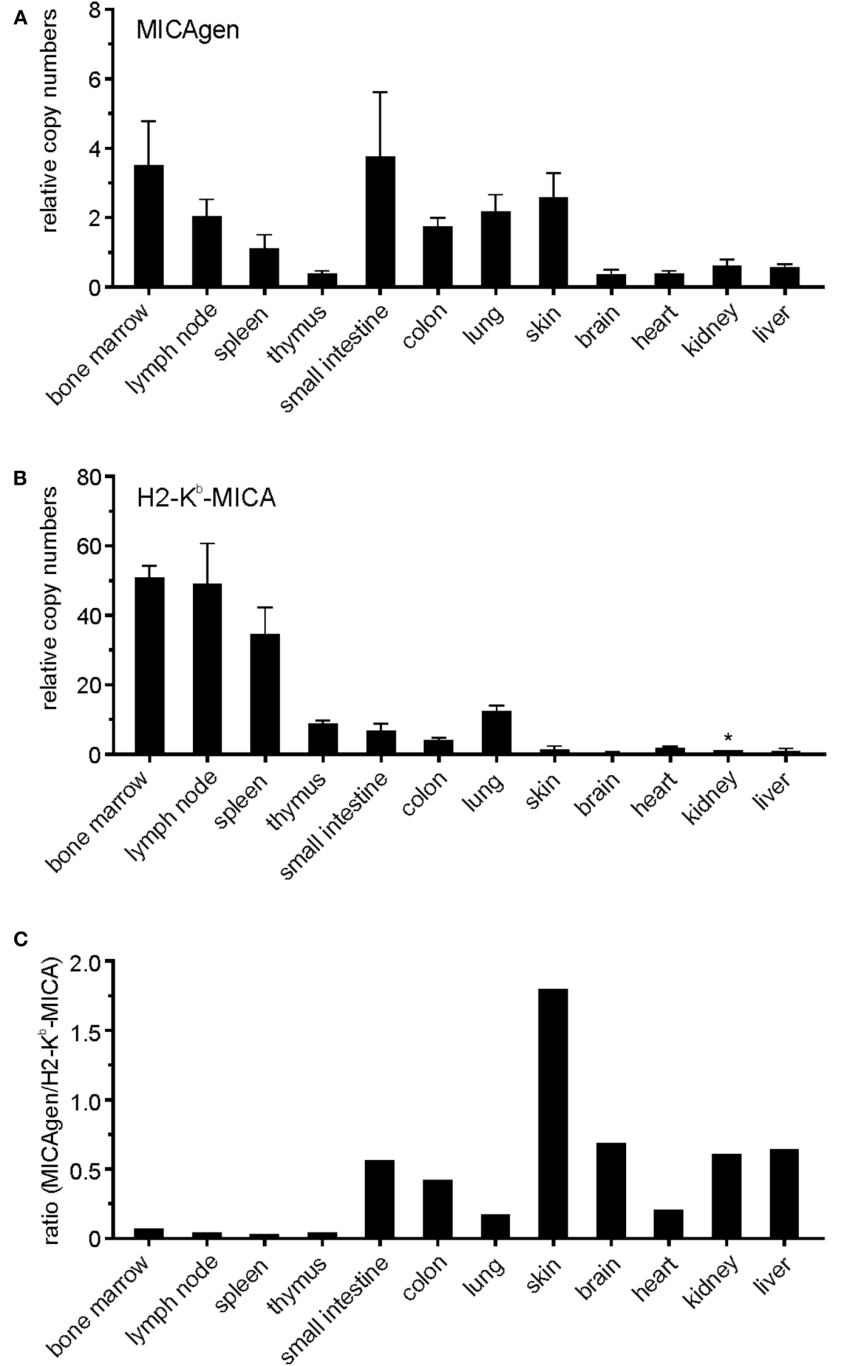
Broad presence of MICA transcripts in various organs of MICAgen mice. **(A,B)** Abundance of MICA transcripts in organs of **(A)** MICAgen mice and **(B)** H2-K^b^-MICA mice. Relative levels of MICA transcripts were determined by qPCR combining primers for exons 3 and 4. Data were normalized to 18S rRNA and arbitrarily set relative to MICA transcript levels in kidneys of H2-K^b^-MICA mice (asterisk) set as 1. Tissues of nontgLM were used as negative control (data not shown). **(C)** Ratios of MICA transcript levels in organs of MICAgen mice set relative to levels in organs of H2-K^b^-MICA mice.

To enable experimental *in vivo* studies on the regulation, function, and disease relevance of MICA, we sought to establish transgenic mice with the complete human MICA gene. To this aim, cosmid MB24/1 was isolated from a cosmid library of human genomic DNA (26) by screening with a human MICA cDNA. A 23.4 kb fragment of cosmid MB24/1 comprising the entire coding sequence of human MICA^*^007 gene (six exons spread across 11.7 kb) plus 10 kb upstream of the translational start site and 1.7 kb downstream of the polyadenylation site (Figure 1) was introduced into the germline of (C57BL/6 × SJL) F1/J hybrid mice. These mice, termed MICAgen mice, were then repeatedly backcrossed to the C57BL/6 background for more than two decades. MICAgen mice present without any obvious abnormalities in phenotype, growth, fertility, and organs and are apparently healthy (not shown). In humans, ample MICA transcripts are present in a wide variety of tissues (32). Similarly, MICA transcripts were broadly detected in all examined organs/tissues of MICAgen mice (Figure 2A) revealing that the human MICA gene can be expressed in rodents, although they do not have MIC genes. MICA transcript levels varied not more than 10-fold between organs of MICAgen mice, with higher relative levels detected for both immune barrier organs (skin, lung, and intestines) and hematopoietic organs (bone marrow, lymph nodes, and spleen) as compared with non-barrier, non-hematopoietic organs (brain, heart, kidney, and liver) (Figure 2A). An exception is the thymus where low MICA transcript levels probably are due to the low MICA expression by T cells (data not shown). In contrast, MICA transcript levels in organs of the previously reported H2-K^b^-MICA transgenic mice expressing the MICA^*^007 cDNA under the control of the MHC class I H2-K^b^ promoter (28) drastically varied (~50-fold to 100-fold) between hematopoietic organs and non-hematopoietic organs (Figure 2B), which reflects the strongly enhanced activity of MHC class I promoters in hematopoietic cells vs. non-hematopoietic cells (33). In line with this, non-hematopoietic barrier organs harboring a high number of immune cells (e.g., the lung and intestines) exhibit intermediate MICA transcript levels (Figure 2B). Importantly, MICA transcript levels in non-hematopoietic organs of MICAgen mice are similar to those of H2-K^b^-MICA mice (Figure 2C) and, hence, in a physiologically relevant range.

### Restricted Tissue Expression of MICA Glycoproteins in MICAgen Mice

**Figure 3:**
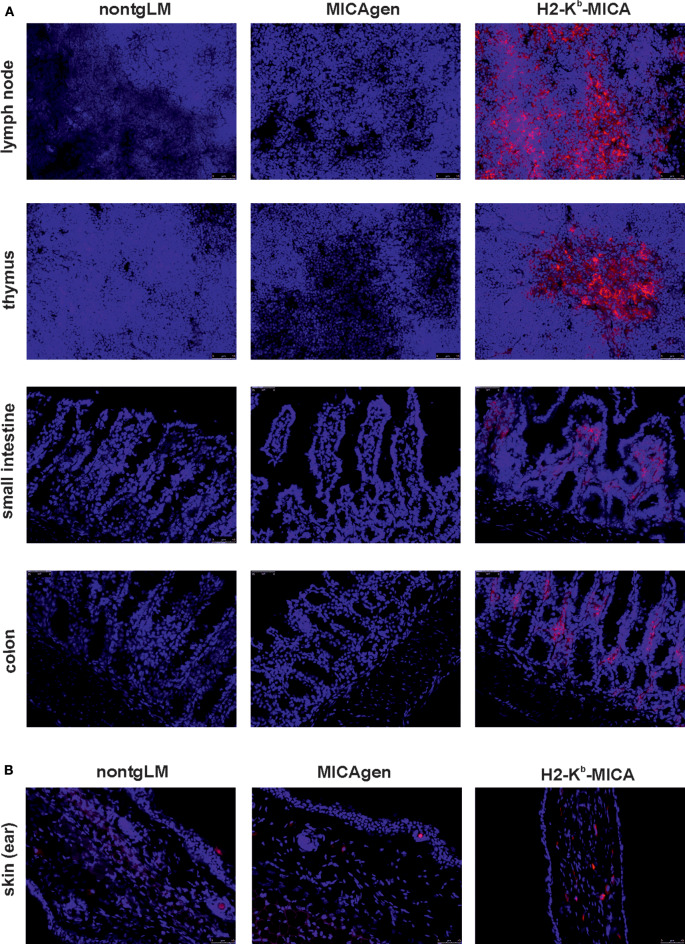
Restricted expression of MICA glycoproteins in MICAgen mice. **(A,B)** MICA glycoproteins are barely detectable in organs of MICAgen mice. Cryosections of **(A)** lymph nodes, thymi, small intestines, and colons and **(B)** skin (ear) of MICAgen mice (middle panels), and for control, of nontgLM (left) and of H2-K^b^-MICA mice (right), were stained with biotinylated anti-MICA/B mAb BAMO1 plus SA-BV421 (red) and counterstained with SYTOX Green (blue) for nuclear staining. Exposure times for SA-BV421 were **(A)** 80 ms and **(B)** 380 ms, respectively. Results are representative for three MICAgen mice and two H2-K^b^-MICA mice analyzed.

In accord with the high abundance of MICA transcripts in hematopoietic organs of H2-K^b^-MICA mice and our previous reports on MICA expression by H2-K^b^-MICA hematopoietic cells (27, 28), we readily detected MICA expression by leukocytes in tissue sections of lymph nodes, thymus, and the large and small intestines of H2-K^b^-MICA mice (Figure 3A). In contrast, MICA molecules could be or not be unambiguously detected in sections of the corresponding organs of MICAgen mice, which presented alike the negative controls from non-transgenic littermates (nontgLM) (Figure 3A). Specifically, no MICA molecules were detectable in the thymus and intestinal epithelium of MICAgen mice, in contrast to reports of MICA expression in the corresponding human tissues (34). In skin, where MICAgen mice and H2-K^b^-MICA mice exhibit similar amounts of MICA transcripts (Figure 2), single MICA-positive cells are present in H2-K^b^-MICA, while such cells could not be unambiguously identified in MICAgen mice (Figure 3B). Altogether, these data are in line with most studies not or only barely detecting MICA molecules in non-malignant, non-diseased human tissues (16, 34) in spite of abundant MICA transcripts. These observations support the notion that the strict post-transcriptional control of MICA glycoprotein expression is maintained in MICAgen mice.

### Restricted Expression of MICA Molecules by MICAgen Splenocytes

**Figure 4:**
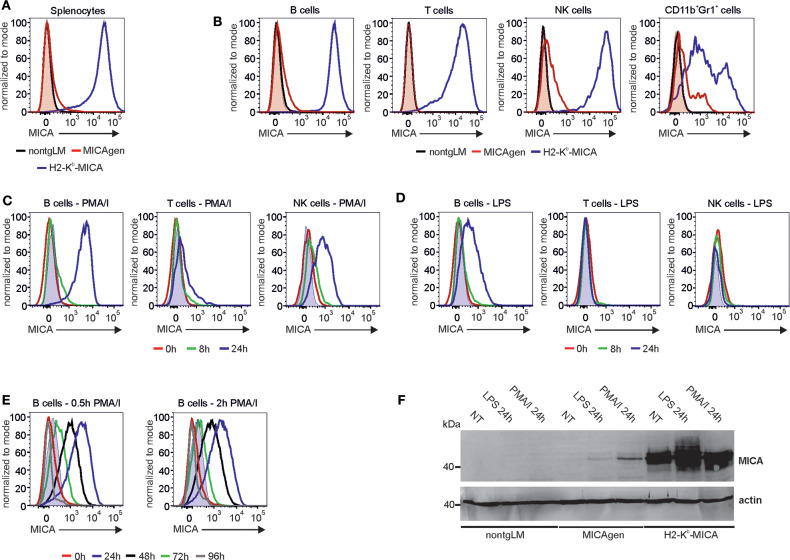
Activation-induced surface expression of MICA molecules on MICAgen splenocytes. **(A)** MICA is barely detectable on total splenocytes of MICAgen mice in contrast to splenocytes of H2-K^b^-MICA mice. Freshly isolated splenocytes were stained for surface MICA and assessed flow cytometry. **(B)** Differential low MICA expression by subsets of MICAgen splenocytes. Freshly isolated splenocytes were stained for surface MICA in addition to various immune markers, and gated for B cells (CD19^+^CD3^−^), T cells (CD19^−^CD3^+^), NK cells (CD11b^+^Gr1^−^NKp46^+^), and myeloid cells (CD11b^+^Gr1^+^), respectively. **(A,B)** MICA stainings (biotinylated BAMO1 plus SA-BV421) of (subgated) splenocytes from MICAgen mice (red line) are overlayed with those of H2-K^b^-MICA mice (blue line) and nontgLM (black line), and negative control stainings (biotinylated irrelevant mouse IgG1 plus SA-BV421) of MICAgen mice (red filled). **(C,D)** Strong induction of surface MICA expression by activated MICAgen splenocytes. Freshly isolated splenocytes of MICAgen mice were treated **(C)** with phorbol myristate acetate (PMA) plus ionomycin (PMA/I) or **(D)** with lipopolysaccharide (LPS) for various times and subsequently MICA cell surface expression of lymphocyte subsets monitored by flow cytometry using biotinylated AMO1 plus SA-BV421. **(C,D)** MICA stainings of subgated splenocytes treated for 0 (red line), 8 (green line), and 24 h (blue line) are overlayed. Negative control stainings (biotinylated irrelevant IgG1 plus SA-BV421) of samples at 24 h of treatment is also overlayed (filled light blue). **(E)** Activation-induced surface MICA expression on MICAgen splenocytes is transient. Freshly isolated splenocytes of MICAgen mice were cultured in presence of PMA/I for either 0.5 (left) or 2 h (right), extensively washed, and subsequently cultured for up to 96 h. MICA surface expression on B cells monitored by flow cytometry using biotinylated AMO1 plus SA-BV421. MICA stainings of B cells before stimulation with PMA/I (red line), and 24 (blue), 48 (black), 72 (green), or 96 h (gray) after begin of stimulation are overlayed. Negative control stainings (biotinylated irrelevant IgG1 plus SA-BV421) of samples at 24 h of treatment are also overlayed (filled blue). **(F)** Activation of MICAgen splenocytes results in *de novo* induced MICA glycoprotein expression. Freshly isolated splenocytes of nontgLM, MICAgen, and H2-K^b^-MICA mice were treated for 24 h with LPS or PMA/I or left untreated (NT), and subsequently, PNGaseF-treated cell lysates were analyzed by immunoblotting with biotinylated BAMO1. Detection of actin as loading control.

As previously reported, splenocytes of H2-K^b^-MICA mice show a strong MICA surface expression (28). In contrast, cell surface MICA was not readily detectable on total splenocytes of MICAgen mice by flow cytometry (Figure 4A). However, when gating on subsets of splenocytes (Figure S1) and using a potent staining procedure, a differential MICA expression pattern was observed: while T cells of MICAgen mice completely are devoid of surface MICA, a marginal MICA surface expression was detected for both B cells and NK cells, which was validated by comparative stainings of non-transgenic splenocytes (Figure 4B). Splenic myeloid cells (CD11b^+^Gr1^+^) showed a substantial MICA expression (Figure 4B) in line with previous reports of MICA expression by human myeloid cells (12, 35). Previous *in vitro* studies reported an induced MICA surface expression upon activation of human leukocytes (11–14, 35). Hence, we wondered whether such an activation-induced MICA expression is recapitulated by MICAgen splenocytes. When potently stimulating MICAgen splenocytes *in vitro* with PMA/ionomycin, surface MICA expression by the various lymphocyte subsets was induced within 24 h, most strongly on B cells, but also on NK cells and T cells (Figure 4C). For B cells, an increase of surface MICA expression was already detectable at 8 h of stimulation and peaked at ~24 h (Figure 4C). Similarly, MICA expression on NK and T cells reached a maximum at 24–48 h after stimulation, although at lower levels (Figure 4C and data not shown). We then addressed induction of MICA expression by a physiologically relevant agent and therefore treated MICAgen splenocytes with the TLR4 ligand LPS. In line with TLR4 expression by mouse B cells, we detected a substantial induction of surface MICA on B cells 24 h after LPS exposure *in vitro*, but not on NK cells and T cells (Figure 4D). Strong MICA up-regulation on activated B cells was validated by staining with both AMO1 and BAMO1, and by staining of equally treated B cells of nontgLM (Figure S2). Next, we assessed permanency of MICA surface expression upon induction. To this aim, MICAgen splenocytes were exposed to a pulse of PMA/ionomycin treatment (0.5 or 2 h) and extensively washed, and cultures continued in the absence of PMA/ionomycin. Such a short pulse was sufficient to achieve similar MICA surface expression levels 24 h later such as in continuous presence of PMA/ionomycin. However, MICA surface expression steadily diminished over the following days and reached background levels 4 days after stimulation (Figure 4E). Immunoblotting of *in vitro*-activated splenocytes demonstrated that induction surface MICA was due to an enhanced *de novo* synthesis of MICA glycoproteins upon stimulation and not due to surfacing of intracellularly retained MICA, since MICA molecules were undetectable in non-stimulated MICAgen splenocytes (Figure 4F). Collectively, these data demonstrate that MICAgen mice are capable to express MICA glycoproteins at the cell surface. But expression of MICA glycoproteins apparently is subjected to a restrictive control, requires induction by activating stimuli such as TLR ligands, and reverts to background levels within a few days. Hence, MICA expression by MICAgen mice recapitulates both the tightly controlled and activation-inducible character of MICA surface expression previously reported for human cells (11–14).

### Soluble MICA in MICAgen Mice

**Figure 5:**
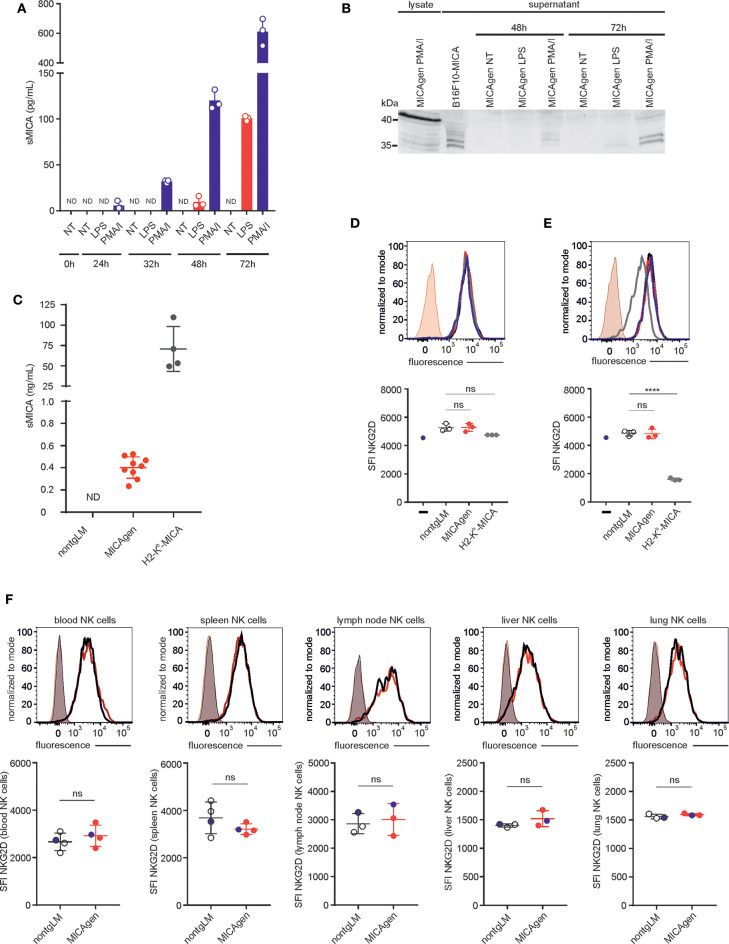
Soluble shed MICA (sMICA) in plasma of MICAgen mice does not down-regulate NKG2D and is derived, at least in part, from hematopoietic cells. **(A,B)** Activated MICAgen splenocytes shed substantial amounts of sMICA. Freshly isolated splenocytes of MICAgen mice were treated *in vitro* with either lipopolysaccharide (LPS) or PMA plus ionomycin (PMA/I), or left untreated (NT) for various times. **(A)** Concentrations of sMICA in the respective culture supernatants were determined by a MICA-specific ELISA. Data from stimulations of splenocytes from three different mice are shown with mean and standard deviation. ND, not detectable. **(B)** Detection of sMICA in PNGaseF-digested culture supernatants of activated splenocytes by immunoblotting using mAb BAMO1. For comparison, lysates of PMA/I-stimulated splenocytes (full-length MICA) and supernatants of B16F10-MICA transductants (sMICA) were analyzed. **(C)** Plasma of MICAgen mice contains substantial amounts (~0.4 ng/ml) of sMICA but markedly less than H2-K^b^-MICA mice (~75 ng/ml). Plasma of nontgLM, as well as of MICAgen and H2-K^b^-MICA mice, was analyzed for sMICA by ELISA. Each symbol represents sMICA levels of an individual mouse. ND, not detectable. Mean and standard deviation are shown. **(D,E)** NKG2D surface expression on NK cells after **(E)** direct co-culture with splenocytes from MICAgen, or H2-K^b^-MICA mice, or nontgLM, respectively, or **(D)** co-culture with such splenocytes in a transwell setting. Carboxyfluorescein succinimidyl ester (CFSE) pre-labeled splenic NK cells from nontgLM **(D)** placed in permeable transwell inserts into cultures of splenocytes from nontgLM, or MICAgen, or H2-K^b^-MICA mice **(E)** or directly co-cultured with splenocytes from nontgLM, or MICAgen, or H2-K^b^-MICA mice, respectively. After 24 h culture, NK cells were stained with anti-NKG2D mAb and analyzed by flow cytometry with gates set on CFSE^+^ CD3^−^NK1.1^+^ cells. Representative stainings of NK cells co-cultured with splenocytes from nontgLM (black line), MICAgen (red line), and H2-K^b^-MICA (gray line) were overlayed. NKG2D expression of NK cells cultured without splenocytes (blue line) is shown for control. Isotype controls stainings (rat IgG1k-BV421) of NK cells co-cultured with MICAgen splenocytes also overlayed. Mean and standard deviation are shown. Each dot represents an individual mouse. One-way ANOVA test was performed (****p < 0.0001, ns = not significant). **(F)** NKG2D expression on blood or tissue NK cells of MICAgen mice is not reduced. Upper panels show representative NKG2D stainings of NK cells isolated from various organs (blood, spleen, lymph node, liver, and lung) of nontgLM (black line) or MICAgen mice (red line), overlayed with isotype control stainings (rat IgG1k-BV421) (shaded). Lower panels show compilation of data from three to four mice per group. Mean and standard deviation are shown. Unpaired *T*-test was performed (ns = not significant). The data shown in upper panels are marked as blue dots.

MICA is proteolytically shed from the cell surface of tumor cells by ADAM proteases, and shed sMICA has been detected in sera of many cancer patients, but also in sera of individuals with acute bacterial infections or cholestasis (22, 31, 36, 37). MICA shedding is considered as an immune escape mechanism of tumors evading NKG2D-mediated tumor immunosurveillance (3, 38) and has recently been successfully targeted in an immunotherapeutic approach using preclinical mouse models (39). Hence, we asked whether MICA shedding and its functional implications for cancer immunosurveillance can also be studied in MICAgen mice. To this aim, we first addressed whether MICA is shed by activated MICAgen splenocytes and therefore measured sMICA in culture supernatants of MICAgen splenocytes treated with either PMA/ionomycin or with LPS using a sMICA-specific sandwich ELISA. Low levels of sMICA were already detectable at 24 h of treatment with PMA/ionomycin, or after 48 h treatment with LPS, which strongly increased during the ensuing 24–48 h (Figure 5A). Immunoblotting confirmed the presence of proteolytically processed sMICA in the culture supernatants, which can be distinguished from cell-associated MICA by its reduced molecular mass (Figure 5B). Next, we asked whether sMICA is also detectable in plasma of MICAgen mice. We consistently detected low but substantial levels of sMICA (~0.4 ng/ml; range: 0.25–0.55 ng/ml) in plasma of unchallenged MICAgen mice by ELISA (Figure 5C). Such sMICA levels are ~100- to 300-fold lower than in plasma of H2-K^b^-MICA mice (28), but roughly in the same range (0.1–2 ng/ml) as reported for humans with malignant and non-malignant diseases (36, 37). Since many studies have implicated elevated sera levels of sMICA in systemic NKG2D down-regulation of cancer patients, we analyzed NKG2D expression by blood NK cells of MICAgen mice. There was no systemic NKG2D down-regulation by sMICA in MICAgen mice, since NKG2D expression levels on peripheral blood NK cells of MICAgen mice were comparable with levels of nontgLM (Figure 5F). NKG2D surface expression levels on NK cells in spleen, lymph nodes, liver, and lung of MICAgen mice were also not different to matched NK cells of nontgLM, indicating that there was no NKG2D down-regulation neither by locally concentrated sMICA nor by any potential membrane-bound MICA (Figure 5F, Figure S3). This is in contrast to NK cells of H2-K^b^-MICA mice where a strong NKG2D down-regulation has previously been reported as a consequence of permanent engagement of ubiquitously expressed membrane-bound MICA (28). To corroborate our *in vivo* findings, we finally addressed the issue of MICA-induced NKG2D down-regulation in MICAgen mice *in vitro*, using MICAgen splenocytes in direct comparison with nontgLM splenocytes and H2-K^b^-MICA splenocytes. In co-cultures with H2-K^b^-MICA splenocytes, NKG2D on NK cells was drastically down-regulated as previously reported (28), while co-cultures with splenocytes from either nontgLM or MICAgen mice did not affect NKG2D surface levels (Figure 5E). When NK cells were spatially separated from splenocytes using a transwell setting, NKG2D surface expression of NK cells was even not altered in cultures with H2-K^b^-MICA splenocytes shedding high amounts of sMICA, confirming previous results that NKG2D in H2-K^b^-MICA splenocytes is down-regulated by membrane-bound MICA and not by the high levels of sMICA (Figure 5D) (28).

### MICAgen Mice Are Immunotolerant for MICA and Functionally Recognize MICA^+^-Syngeneic Mouse Tumors in an NKG2D-Dependent Manner

**Figure 6:**
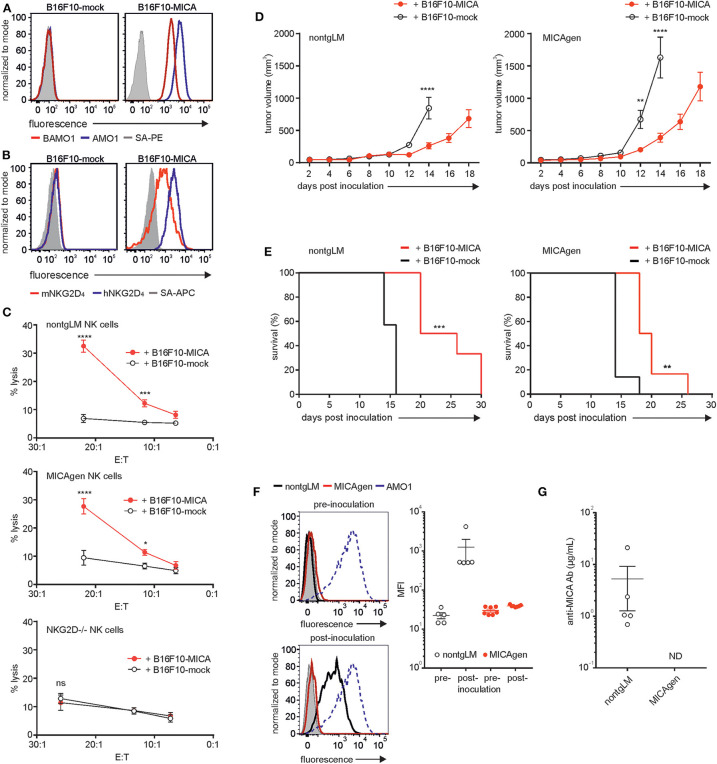
MICAgen mice are immunotolerant for the human xenoantigen MICA and functionally recognize MICA-expressing tumors in an NKG2D-dependent manner. **(A,B)** MICA surface expression and NKG2D ligation by B16F10-MICA tumor cells. B16F10-MICA and B16F10-mock transductants were stained **(A)** with either biotinylated AMO1 (blue line) or biotinylated BAMO1 (red line) plus SA-PE or **(B)** with recombinant human NKG2D (blue line) or mouse NKG2D (red line) tetramerized with SA-APC. Control stainings with SA-conjugates only (gray shaded). **(C)** MICA is functionally recognized by mouse NK cells *via* NKG2D. Chromium-labeled B16F10-MICA*001 or B16F10-mock cells were co-incubated with freshly purified, poly(I:C) pre-activated splenic NK cells from nontgLM (upper), MICAgen (middle), and NKG2D-deficient mice (lower) for 4 h at 37°C and % cytolysis calculated by analyzing supernatants. Means and standard deviation of triplicates are shown. Two-way ANOVA test was performed (****p < 0.0001, ***p < 0.0002, *p < 0.0332, ns = not significant). **(D,E)** Tumor-associated MICA improves survival of tumor-bearing MICAgen mice. **(D)** Impaired growth of B16F10-MICA*001 compared with B16F10-mock tumors in both nontgLM mice (left) and in MICAgen mice (right). Mean and standard error are shown. Two-way ANOVA test was performed (****p < 0.0001, **p < 0.0021). **(E)** Improved survival mice with B16F10-MICA*001 tumors as compared with B16F10-mock tumors. 1 × 10^4^ B16F10-MICA*001 or B16F10-mock tumor cells were inoculated subcutaneously, and tumor growth was monitored by measuring tumor volume. Log rank (Mantel–Cox) test was performed (***p < 0.0002, **p < 0.002). **(F,G)** MICAgen mice are immunotolerant for MICA. **(F)** Plasma of MICAgen mice or nontgLM was analyzed for anti-MICA antibodies prior to inoculation with B16F10-MICA cells (pre) and after euthanasia due to the size of B16F10-MICA tumors (post). Presence of MICA-specific antibodies in mouse plasma was assessed by flow cytometry of C1R-MICA*001 incubated with mouse plasma (diluted 1:50) and fluorochrome-conjugated anti-mouse-Ig antibodies. (**F**, left) Representative stainings of C1R-MICA*001 cells with (pre) and (post) plasma from nontgLM and MICAgen mice. Control stainings with AMO1 (stippled) and normal mouse plasma (gray shaded). (**F**, right) Compilation of MFI of C1R-MICA stainings with plasma from >5 tumor-bearing mice per group. Means and standard error are shown. **(G)** Concentrations of anti-MICA antibodies in plasma of tumor-bearing mice were determined by a direct ELISA. Plasma of MICAgen or nontgLM mice was analyzed after euthanasia due to growth of B16F10-MICA tumors. Mean and standard error are shown. ND = not detectable (detection limit ~ 1 ng/ml).

Therapeutic strategies targeting human MICA molecules, for example, by employing MICA-specific antibodies in cancer immunotherapy, are increasingly getting into the focus of current translational research. *In vivo* evaluation of the therapeutic efficacy of such antibodies in syngeneic and immunocompetent mouse tumor models requires inoculation of mouse tumor cells ectopically expressing MICA. However, the xenoantigen MICA provokes strong humoral and cellular responses of the adaptive immune system confounding the data on therapeutic efficacy (39). Hence, in such a scenario, MICAgen mice may be of major advantage, as they expectedly exhibit immunotolerance for MICA. To assess MICAgen mice for both functional NKG2D-mediated immunorecognition of MICA^+^-tumors and for immunotolerance toward the xenoantigen MICA, we used B16F10 melanoma cells ectopically expressing human MICA^*^001. B16F10-MICA cells brightly express MICA at the cell surface, which ligates both human and mouse NKG2D (Figures 6A,B). In contrast, control-transduced B16F10-mock cells do not bind mouse NKG2D, as B16F10 cell do not express mouse NKG2D ligands (Figure 6B). MICA expression rendered B16F10-MICA cells strongly susceptible to cytolysis by purified splenic NK cells from both nontgLM and MICAgen mice, but not from NKG2D-deficient mice (Figure 6C), demonstrating unimpaired NKG2D-mediated immunorecognition of tumor-associated human MICA by MICAgen NK cells. A low number (1 × 10^4^) of B16F10-MICA or B16F10-mock cells (in Matrigel) was subcutaneously inoculated into either MICAgen mice or nontgLM and tumor growth monitored. Growth of B16F10-MICA tumors as compared with B16F10-mock tumors was markedly delayed in both nontgLM and in MICAgen mice (Figure 6D). Accordingly, MICA expression by B16F10 tumors significantly improved survival of both nontgLM and MICAgen mice (Figure 6E), indicating that MICAgen mice mount an effective immune response against MICA-expressing tumors. However, such an anti-MICA-directed immune response could be mediated not only *via* NKG2D but also by humoral and cellular immune responses against the xenoantigen MICA. Therefore, we asked whether MICA-expressing B16F10 tumors raise adaptive anti-MICA responses that may confound these results. In fact, all nontgLM bearing B16F10-MICA tumors raised a strong humoral anti-MICA response within 10–14 days post tumor inoculation, as their plasma contained high titers of MICA-specific antibodies up to 20 μg/ml (Figures 6F,G). In contrast, no anti-MICA antibodies were detectable in plasma of any of the B16F10-MICA tumor-bearing MICAgen mice testifying their immunotolerance for MICA (Figures 6F,G). Hence, MICAgen mice are immunotolerant for MICA and respond to MICA-expressing tumors primarily in an NKG2D-dependent manner. These findings show that MICAgen mice are more suited for immunotherapeutic studies targeting MICA than conventional mice.

## Discussion

About 25 years after the description of the human MICA gene (40) and ~20 years after the functional characterization of MICA as stress-inducible ligand of NKG2D (1), we recognize an as of yet unmet need to establish a *bona fide* mouse model for studies of MICA expression and function *in vivo*. Although numerous *in vitro* studies or correlative analyses have investigated a role of MICA in the regulation of immune responses, in the recognition and elimination of tumor or virus-infected cells, and in the pathogenesis of various autoimmune disorders, conclusions from many of these studies are limited by the lack of corroborative *in vivo* analyses. We believe that the mouse model presented here, in spite of its obvious deficits, will be helpful to advance our understanding of the human NKG2D/NKG2DL system by enabling *in vivo* studies of MICA expression and function. For example, understanding regulation of MICA expression is key for its function as a “kill-me” signal for NKG2D-expressing cytotoxic lymphocytes. Our data presented here suggest that MICA expression in MICAgen mice is subjected to a similarly restrictive control as reported for humans: although we abundantly detected MICA transcripts in a wide variety of organs, we could not detect MICA molecules *in situ* paralleling most reports on a restricted MICA expression by healthy human tissues (16, 34). However, we cannot exclude that our *in situ* detection methodology may have failed to detect very low levels of MICA expression. With the use of more sensitive flow cytometry, we detected some residual MICA expression on splenic subsets such as NK cells and myeloid cells *ex vivo*. Immunoblotting of freshly isolated splenocytes confirmed that expression of MICA glycoproteins is restricted in MICAgen mice and only becomes detectable upon activation of splenocytes. Specifically, we find that splenic B cells drastically up-regulate MICA upon activation, an observation that has not yet been reported for humans and needs to be addressed. In contrast, several studies had reported up-regulation of MICA by activated human myeloid cells, for example, upon treatment with TLR ligands, or by activated human T cells (11–14). Hence, regulation and functional consequences of activation-induced up-regulation of MICA can be studied using MICAgen mice. MICAgen mice do not only provide unlimited material for *in vitro* studies of MICA induction but also allow to study MICA regulation and induction *in vivo* using genetically-altered mouse models, treatment with small molecules or carcinogens, or by inducing autoimmune diseases. In the context of cancer immunology, it will be of particular interest to address how MICA expression is induced and regulated during malignant transformation, how MICA expression tunes anti-tumor immune response, and how tumor mediated shedding of MICA, and sMICA itself impacts on the disease course. We also confirmed the immunological supposition that MICAgen mice are immunotolerant for MICA, which renders them a valuable tool for *in vivo* experiments with MICA-expressing tumor cells and/or MICA-targeting antibodies. Notably, there are previous reports of anti-MICA antibodies generated in cancer patients in the course of disease and/or treatment. Melanoma and non-small cell lung carcinoma patients vaccinated with irradiated, autologous granulocyte-macrophage colony-stimulating factor (GM-CSF)-secreting tumor cells and partially treated with anti-CTLA-4 antibodies generated high levels of anti-MICA antibodies, which were ascribed anti-tumor effects, for example, by neutralizing sMICA, increasing NKG2D expression, and complement-mediated tumor lysis (41). In several patients with monoclonal gammopathy of undetermined significance (MGUS), characterized by a restricted clonal expansion of antibody-secreting plasma cells in the bone marrow, the occurrence of anti-MICA antibodies was reported paralleling a pronounced MICA expression on the expanding plasma cells (42). Of note, patients with multiple myeloma (MM), which occasionally evolves from MGUS, show lower MICA surface expression on MM cells and lower anti-MICA antibody levels but strongly increased sMICA levels, which may foster disease progression (42). It will be of considerable interest to cross MICAgen mice with mouse models developing MM-like diseases (43) to thoroughly investigate the mechanisms of MICA induction and shedding by malignant cells, the emergence of anti-MICA antibodies, and their therapeutic benefit for the course of disease.

Our characterization of MICAgen mice also resulted in a couple of observations that may importantly contribute to our understanding of MICA biology and function. Since the first reports on MICA shedding and sMICA in sera of cancer patients (31, 44), there have been dozens of studies with conflicting data on the effects of sMICA molecules on NKG2D surface expression (45–47). While, numerous studies claimed that sMICA molecules directly down-regulate NKG2D on NK cells and T cells, other studies found no evidence for an NKG2D down-regulation by sMICA (20, 47). Here, we find that NKG2D is neither systemically down-regulated on peripheral blood NK cells of MICAgen mice in spite of substantial amounts of sMICA plasma levels comparable with levels detected in many cancer patients (36, 37) nor on NK cells in various organs of MICAgen mice where sMICA levels may be locally concentrated (e.g., in spleen or lymph nodes). We further corroborate our previous findings (28) that membrane-bound MICA of H2-K^b^-MICA splenocytes down-regulates NKG2D upon direct cellular interaction with NK cells, while there is no down-regulation in a transwell setting where only shed sMICA acts on NK cells. Collectively, all our data strongly argue against an NKG2D down-regulation by shed sMICA, while some previous observations on NKG2D down-regulation in cancer patients may be attributable to MICA embedded in exosomes or other tumor-derived vesicles or by other tumor-derived soluble factors such as TGF-β (20, 45, 46). Further, our data suggest that sMICA in plasma of MICAgen mice is, at least in part, derived from hematopoietic cells that up-regulated and shed MICA upon activation (Figure 5). Such sMICA may differ in its effects on NKG2D modulation from tumor-released MICA, especially when embedded in exosomes (45, 48, 49). Of note, activation-induced surface MICA is vanishing over several days past activation, and it will be interesting to determine the mechanisms not only underlying MICA induction but also underlying the reversal of MICA surface expression. Also for this purpose, a robust mouse model such as MICAgen mice amenable to genetic experiments will be valuable.

However, since MICA is highly polymorphic with more than 150 alleles reported to date encoding for more than 90 different MICA proteins (50, 51), certain results obtained from studies of this mouse model expressing MICA^*^007 may not be generally applicable to all MICA variants: these limitations may specifically relate to functional *in vivo* studies with regard to MICA allelic variants substantially differing from MICA^*^007 in the affinity for NKG2D (such as MICA^*^004) (5, 52, 53) or in the membrane localization and mode of release (such as MICA^*^008) (54, 55).

Taken together, we here provide a thorough characterization of MICAgen mice, which apparently express the human MICA gene in a strictly controlled but inducible manner very much alike that of humans. Therefore, these mice will be helpful in the context of (i) translational and therapeutic research requiring MICA-immunotolerant mice, (ii) studies on the regulation and induction of MICA expression, and (iii) studies on the functional relevance of MICA in the context of immune responses to infections, tumors, and autoimmune diseases.

## Data Availability Statement

All datasets generated for this study are included in the article/Supplementary Material.

## Ethics Statement

The animal study was reviewed and approved by Regierungspräsidium Darmstadt.

## Author Contributions

YK and CB performed the experiments and analyzed the data. MB provided some reagents and critical input. AS conceptualized the study, designed the experiments, and wrote the manuscript with the support of YK and CB.

## Conflict of Interest

MICAgen mice are currently used to preclinically evaluate MICA-targeted immunotherapies. MICAgen mice are available upon licensing. The authors declare that the research was conducted in the absence of any commercial or financial relationships that could be construed as a potential conflict of interest.

## References

[1] Bauer S Groh V Wu J Steinle A Phillips JH Lanier LL Activation of NK cells and T cells by NKG2D, a receptor for stress-inducible MICA. Science. (1999) 285:727–9. 10.1126/science.285.5428.727 10426993

[2] Waldhauer I Steinle A. NK cells and cancer immunosurveillance. Oncogene. (2008) 27:5932–43. 10.1038/onc.2008.267 18836474

[3] Lazarova M Steinle A. The NKG2D axis: an emerging target in cancer immunotherapy. Expert Opin Ther Targets. (2019) 23:281–94. 10.1080/14728222.2019.1580693 30732494

[4] Ullrich E Koch J Cerwenka A Steinle A. New prospects on the NKG2D/NKG2DL system for oncology. Oncoimmunology. (2013) 2:e26097. 10.4161/onci.26097 24353908 PMC3862635

[5] Steinle A Li P Morris DL Groh V Lanier LL Strong RK. Interactions of human NKG2D with its ligands MICA, MICB, and homologs of the mouse RAE-1 protein family. Immunogenetics. (2001) 53:279–87. 10.1007/s002510100325 11491531

[6] Li P Morris DL Willcox BE Steinle A Spies T Strong RK. Complex structure of the activating immunoreceptor NKG2D and its MHC class I-like ligand MICA. Nat. Immunol. (2001) 2:443–51. 10.1038/87757 11323699

[7] Muller S Zocher G Steinle A Stehle T. Structure of the HCMV UL16-MICB complex elucidates select binding of a viral immunoevasin to diverse NKG2D ligands. PLoS. Pathog. (2010) 6:e1000723. 10.1371/journal.ppat.1000723 20090832 PMC2797645

[8] Sester M Koebernick K Owen D Ao M Bromberg Y May E. Conserved amino acids within the adenovirus 2 E3/19K protein differentially affect downregulation of MHC class I and MICA/B proteins. J Immunol. (2010) 184:255–67. 10.4049/jimmunol.0902343 19949079

[9] Welte SA Sinzger C Lutz SZ Singh-Jasuja H Sampaio KL Eknigk U. Selective intracellular retention of virally induced NKG2D ligands by the human cytomegalovirus UL16 glycoprotein. Eur J Immunol. (2003) 33:194–203. 10.1002/immu.200390022 12594848

[10] Miletic A Krmpotic A Jonjic S. The evolutionary arms race between NK cells and viruses: who gets the short end of the stick? Eur J Immunol. (2013) 43:867–77. 10.1002/eji.201243101 23440773

[11] Cerboni C Ardolino M Santoni A Zingoni A. Detuning CD8+ T lymphocytes by down-regulation of the activating receptor NKG2D: role of NKG2D ligands released by activated T cells. Blood. (2009) 113:2955–64. 10.1182/blood-2008-06-165944 19124832

[12] Kloss M Decker P Baltz KM Baessler T Jung G Rammensee HG. Interaction of monocytes with NK cells upon Toll-like receptor-induced expression of the NKG2D ligand MICA. J. Immunol. (2008) 181:6711–9. 10.4049/jimmunol.181.10.6711 18981088

[13] Molinero LL Fuertes MB Rabinovich GA Fainboim L Zwirner NW. Activation-induced expression of MICA on T lymphocytes involves engagement of CD3 and CD28. J Leukoc Biol. (2002) 71:791–7. 10.1189/jlb.71.5.791 11994503

[14] Trembath AP Markiewicz MA. More than decoration: roles for natural killer group 2 member d ligand expression by immune cells. Front Immunol. (2018) 9:231. 10.3389/fimmu.2018.00231 29483917 PMC5816059

[15] Babic M Romagnani C. The role of natural killer group 2, member d in chronic inflammation and autoimmunity. Front Immunol. (2018) 9:1219. 10.3389/fimmu.2018.01219 29910814 PMC5992374

[16] Groh V Rhinehart R Secrist H Bauer S Grabstein KH Spies T. Broad tumor-associated expression and recognition by tumor-derived gamma delta T cells of MICA and MICB. Proc Natl Acad Sci USA. (1999) 96:6879–84. 10.1073/pnas.96.12.6879 10359807 PMC22010

[17] Pende D Cantoni C Rivera P Vitale M Castriconi R Marcenaro S. Role of NKG2D in tumor cell lysis mediated by human NK cells: cooperation with natural cytotoxicity receptors and capability of recognizing tumors of nonepithelial origin. Eur J Immunol. (2001) 31:1076–86. 10.1002/1521-4141(200104)31:4<1076::AID-IMMU1076>3.0.CO;2-Y 11298332

[18] Friese MA Wischhusen J Wick W Weiler M Eisele G Steinle A. RNA interference targeting transforming growth factor-beta enhances NKG2D-mediated antiglioma immune response, inhibits glioma cell migration and invasiveness, and abrogates tumorigenicity *in vivo*. Cancer Res. (2004) 64:7596–603. 10.1158/0008-5472.CAN-04-1627 15492287

[19] Dhar P Wu JD. NKG2D and its ligands in cancer. Curr Opin Immunol. (2018) 51:55–61. 10.1016/j.coi.2018.02.004 29525346 PMC6145810

[20] Lazarova M Steinle A. Impairment of NKG2D-mediated tumor immunity by TGF-beta. Front Immunol. (2019) 10:2689. 10.3389/fimmu.2019.02689 31803194 PMC6873348

[21] Stern-Ginossar N Mandelboim O. An integrated view of the regulation of NKG2D ligands. Immunology. (2009) 128:1–6. 10.1111/j.1365-2567.2009.03147.x 19689730 PMC2747133

[22] Waldhauer I Goehlsdorf D Gieseke F Weinschenk T Wittenbrink M Ludwig A. Tumor-associated MICA is shed by ADAM proteases. Cancer Res. (2008) 68:6368–76. 10.1158/0008-5472.CAN-07-6768 18676862

[23] Eisele G Wischhusen J Mittelbronn M Meyermann R Waldhauer I Steinle A. TGF-beta and metalloproteinases differentially suppress NKG2D ligand surface expression on malignant glioma cells. Brain. (2006) 129:2416–25. 10.1093/brain/awl205 16891318

[24] Cerwenka A Baron JL Lanier LL. Ectopic expression of retinoic acid early inducible-1 gene (RAE-1) permits natural killer cell-mediated rejection of a MHC class I-bearing tumor *in vivo*. Proc Natl Acad Sci USA. (2001) 98:11521–6. 10.1073/pnas.201238598 11562472 PMC58762

[25] Diefenbach A Jensen ER Jamieson AM Raulet DH. Rae1 and H60 ligands of the NKG2D receptor stimulate tumour immunity. Nature. (2001) 413:165–71. 10.1038/35093109 11557981 PMC3900321

[26] Spies T Blanck G Bresnahan M Sands J Strominger JL. A new cluster of genes within the human major histocompatibility complex. Science. (1989) 243:214–7. 10.1126/science.2911734 2911734

[27] Koch C Kim Y Zoller T Born C Steinle A. Chronic NKG2D engagement *in vivo* differentially impacts NK cell responsiveness by activating NK receptors. Front Immunol. (2017) 8:1466. 10.3389/fimmu.2017.01466 29163533 PMC5675847

[28] Wiemann K Mittrucker HW Feger U Welte SA Yokoyama WM Spies T. Systemic NKG2D down-regulation impairs NK and CD8 T cell responses *in vivo*. J Immunol. (2005) 175:720–9. 10.4049/jimmunol.175.2.720 16002667

[29] Zafirova B Mandaric S Antulov R Krmpotic A Jonsson H Yokoyama WM. Altered NK cell development and enhanced NK cell-mediated resistance to mouse cytomegalovirus in NKG2D-deficient mice. Immunity. (2009) 31:270–82. 10.1016/j.immuni.2009.06.017 19631564 PMC2782462

[30] Salih HR Antropius H Gieseke F Lutz SZ Kanz L Rammensee HG. Functional expression and release of ligands for the activating immunoreceptor NKG2D in leukemia. Blood. (2003) 102:1389–96. 10.1182/blood-2003-01-0019 12714493

[31] Salih HR Rammensee HG Steinle A. Cutting edge: down-regulation of MICA on human tumors by proteolytic shedding. J Immunol. (2002) 169:4098–102. 10.4049/jimmunol.169.8.4098 12370336

[32] Schrambach S Ardizzone M Leymarie V Sibilia J Bahram S. *In vivo* expression pattern of MICA and MICB and its relevance to auto-immunity and cancer. PLoS ONE. (2007) 2:e518. 10.1371/journal.pone.0000518 17565371 PMC1885219

[33] David-Watine B Israel A Kourilsky P. The regulation and expression of MHC class I genes. Immunol Today. (1990) 11:286–92. 10.1016/0167-5699(90)90114-O 1698378

[34] Groh V Bahram S Bauer S Herman A Beauchamp M Spies T. Cell stress-regulated human major histocompatibility complex class I gene expressed in gastrointestinal epithelium. Proc Natl Acad Sci USA. (1996) 93:12445–50. 10.1073/pnas.93.22.12445 8901601 PMC38011

[35] Nedvetzki S Sowinski S Eagle RA Harris J Vely F Pende D. Reciprocal regulation of human natural killer cells and macrophages associated with distinct immune synapses. Blood. (2007) 109:3776–85. 10.1182/blood-2006-10-052977 17218381

[36] Holdenrieder S Eichhorn P Beuers U Samtleben W Stieber P Nagel D. Soluble NKG2D ligands in hepatic autoimmune diseases and in benign diseases involved in marker metabolism. Anticancer Res. (2007) 27:2041–5. 17649819

[37] Holdenrieder S Stieber P Peterfi A Nagel D Steinle A Salih HR. Soluble MICA in malignant diseases. Int J Cancer. (2006) 118:684–7. 10.1002/ijc.21382 16094621

[38] Fernandez-Messina L Reyburn HT Vales-Gomez M. Human NKG2D-ligands: cell biology strategies to ensure immune recognition. Front Immunol. (2012) 3:299. 10.3389/fimmu.2012.00299 23056001 PMC3457034

[39] de Andrade LF Tay RE Pan D Luoma AM Ito Y Badrinath S. Antibody-mediated inhibition of MICA and MICB shedding promotes NK cell-driven tumor immunity. Science. (2018) 359:1537–42. 10.1126/science.aao0505 29599246 PMC6626532

[40] Bahram S Bresnahan M Geraghty DE Spies T. A second lineage of mammalian major histocompatibility complex class I genes. Proc Natl Acad Sci USA. (1994) 91:6259–63. 10.1073/pnas.91.14.6259 8022771 PMC44180

[41] Jinushi M Hodi FS Dranoff G. Therapy-induced antibodies to MHC class I chain-related protein A antagonize immune suppression and stimulate antitumor cytotoxicity. Proc Natl Acad Sci USA. (2006) 103:9190–5. 10.1073/pnas.0603503103 16754847 PMC1482588

[42] Jinushi M Vanneman M Munshi NC Tai YT Prabhala RH Ritz J. MHC class I chain-related protein A antibodies and shedding are associated with the progression of multiple myeloma. Proc Natl Acad Sci USA. (2008) 105:1285–90. 10.1073/pnas.0711293105 18202175 PMC2234130

[43] Garrett IR Dallas S Radl J Mundy GR. A murine model of human myeloma bone disease. Bone. (1997) 20:515–20. 10.1016/S8756-3282(97)00056-2 9177864

[44] Groh V Wu J Yee C Spies T. Tumour-derived soluble MIC ligands impair expression of NKG2D and T-cell activation. Nature. (2002) 419:734–8. 10.1038/nature01112 12384702

[45] Clayton A Mitchell JP Court J Linnane S Mason MD Tabi Z. Human tumor-derived exosomes down-modulate NKG2D expression. J Immunol. (2008) 180:7249–58. 10.4049/jimmunol.180.11.7249 18490724

[46] Crane CA Han SJ Barry JJ Ahn BJ Lanier LL Parsa AT. TGF-beta downregulates the activating receptor NKG2D on NK cells and CD8+ T cells in glioma patients. Neuro Oncol. (2010) 12:7–13. 10.1093/neuonc/nop009 20150362 PMC2940557

[47] Salih HR Holdenrieder S Steinle A. Soluble NKG2D ligands: prevalence, release, and functional impact. Front Biosci. (2008) 13:3448–56. 10.2741/2939 18508446

[48] Ashiru O Boutet P Fernandez-Messina L Aguera-Gonzalez S Skepper JN Vales-Gomez M. Natural killer cell cytotoxicity is suppressed by exposure to the human NKG2D ligand MICA^*^008 that is shed by tumor cells in exosomes. Cancer Res. (2010) 70:481–9. 10.1158/0008-5472.CAN-09-1688 20068167 PMC2817492

[49] Lopez-Cobo S Campos-Silva C Moyano A Oliveira-Rodriguez M Paschen A Yanez-Mo M. Immunoassays for scarce tumour-antigens in exosomes: detection of the human NKG2D-Ligand, MICA, in tetraspanin-containing nanovesicles from melanoma. J Nanobiotechnol. (2018) 16:47. 10.1186/s12951-018-0372-z 29720199 PMC5932892

[50] Robinson J Halliwell JA Hayhurst JD Flicek P Parham P Marsh SG. The IPD and IMGT/HLA database: allele variant databases. Nucleic Acids Res. (2015) 43 D423–31. 10.1093/nar/gku1161 25414341 PMC4383959

[51] Klussmeier A Massalski C Putke K Schafer G Sauter J Schefzyk D. High-Throughput MICA/B genotyping of over two million samples: workflow and allele frequencies. Front Immunol. (2020) 11:314. 10.3389/fimmu.2020.00314 32153595 PMC7047279

[52] Xu B Pizarro JC Holmes MA McBeth C Groh V Spies T. Crystal structure of a gammadelta T-cell receptor specific for the human MHC class I homolog MICA. Proc Natl Acad Sci USA. (2011) 108:2414–9. 10.1073/pnas.1015433108 21262824 PMC3038733

[53] Isernhagen A Malzahn D Bickeboller H Dressel R. Impact of the MICA-129Met/Val dimorphism on NKG2D-Mediated biological functions and disease risks. Front Immunol. (2016) 7:588. 10.3389/fimmu.2016.00588 28018354 PMC5149524

[54] Eleme K Taner SB Onfelt B Collinson LM McCann FE Chalupny NJ. Cell surface organization of stress-inducible proteins ULBP and MICA that stimulate human NK cells and T cells via NKG2D. J Exp Med. (2004) 199:1005–10. 10.1084/jem.20032194 15051759 PMC2211882

[55] Aguera-Gonzalez S Gross CC Fernandez-Messina L Ashiru O Esteso G Hang HC. Palmitoylation of MICA, a ligand for NKG2D, mediates its recruitment to membrane microdomains and promotes its shedding. Eur J Immunol. (2011) 41:3667–76. 10.1002/eji.201141645 21928280 PMC3709245

