# Supplementary material for: MICAgen Mice Recapitulate the Highly Restricted but Activation-Inducible Expression of the Paradigmatic Human NKG2D Ligand MICA
Source: Front Immunol. 2020 Jun 4;11:960. doi: 10.3389/fimmu.2020.00960 (PMC7287395; doi:10.3389/fimmu.2020.00960)
Supplement: Supplementary file 1 [file Data_Sheet_1.PDF]

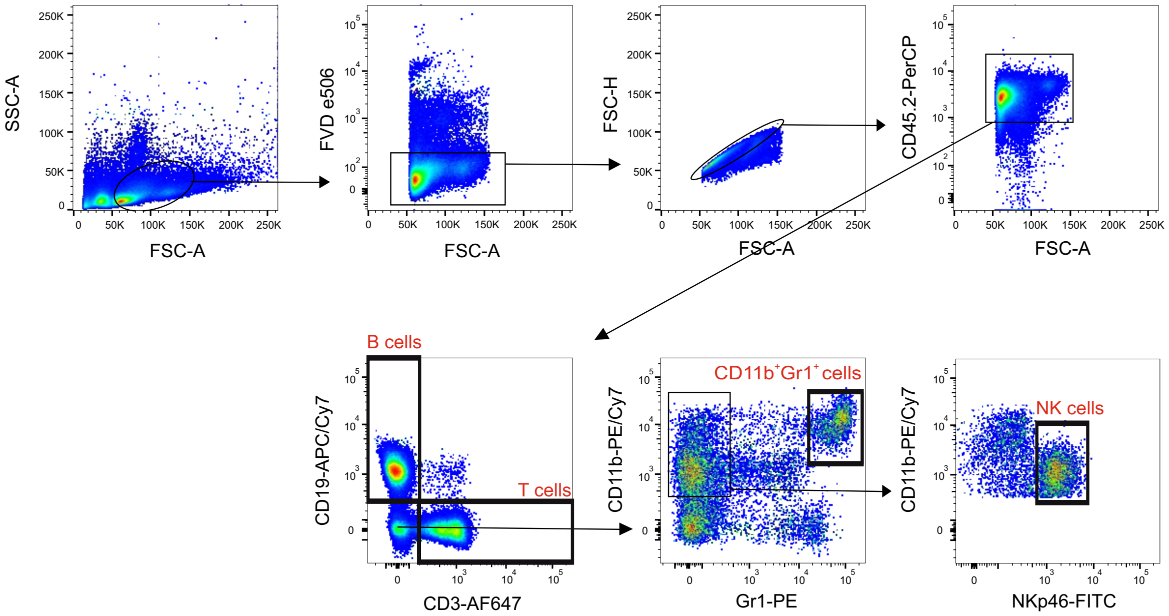

Supplementary Figure S1: Gating strategy for subpopulations of splenic immune cells. Single cell suspensions of mouse splenocytes were stained with various antibodies and analyzed by flow cytometry using a FACSCanto II. Single living (FVDe506-negative) $CD45^+$ cells were subgated for: B cells ($CD19^+CD3^-$), T cells ($CD19^-CD3^+$), myeloid cells ($CD19^-CD3^-CD11b^+Gr1^+$), and NK cells ($CD19^-CD3^-CD11b^+Gr1^-NKp46^+$) as depicted.

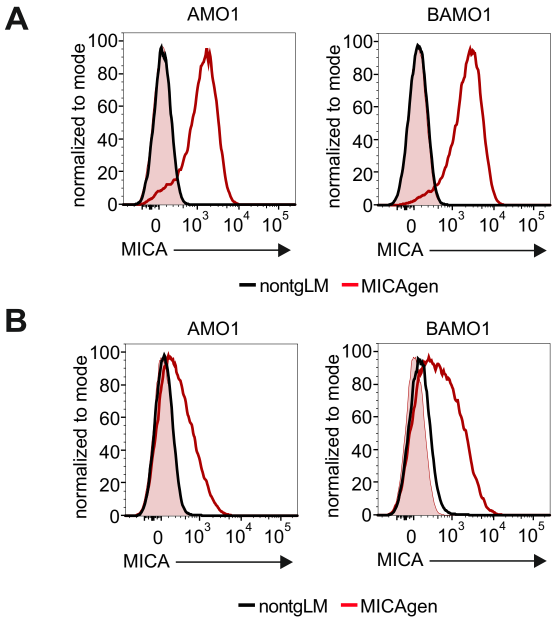

Supplementary Figure S2: Strong activation-induced MICA surface expression on splenic B cells. (A, B) Freshly isolated splenocytes of MICAgen and nontgLM mice were treated (A) with a combination of 50 ng/ml PMA and 1 µM ionomycin (PMA/I) or (B) with with 10 µg/ml lipopolysaccharide (LPS) for 24 h *in vitro* and, subsequently, stained with biotinylated anti-MICA mAb AMO1 plus SA-BV421 (left) or biotinylated anti-MICA/B mAb BAMO1 plus SA-BV421 (right) or biotinylated irrelevant IgG1 plus SA-BV421 (control stainings). MICA cell surface stainings were detected by flow cytometry. MICA stainings of subgated splenic B cells from MICAgen mice (red line) or from nontgLM (black line) are overlayed together with negative control stainings of MICAgen splenic B cells (filled red).

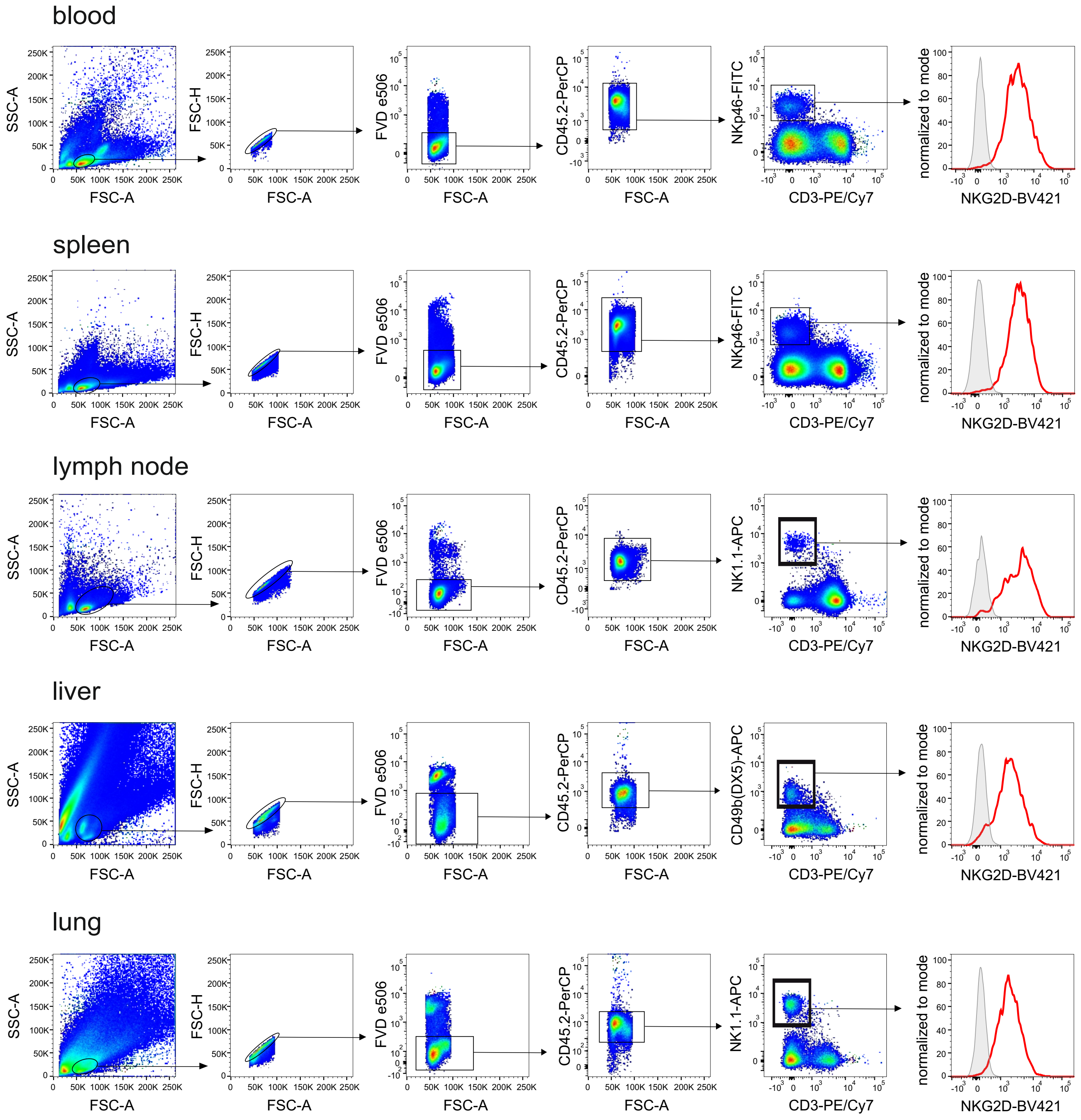

Supplementary Figure S3: Gating strategy for NK cells of mouse organs. Single cell suspensions obtained by passing minced mouse organs (spleen, lymph nodes, liver, lung) or mouse blood cells were stained with various antibodies and analyzed by flow cytometry using a FACSCanto II. Single living (FVDe506 negative) CD45$^+$ cells were subgated for NK cells by additional gating either on CD3$^-$NKp46$^+$ cells (blood, spleen), or on CD3$^-$NK1.1$^+$ cells (lymph node, lung) or on CD3$^-$CD49b$^+$ cells (liver) as depicted.
